# Military responses to COVID-19, emerging trends in global civil-military engagements

**DOI:** 10.1017/S0260210521000048

**Published:** 2021-01-21

**Authors:** Fawzia Gibson-Fall

**Affiliations:** School of Politics and International Relations, Queen Mary University of London, United Kingdom

**Keywords:** Militaries, Global Health, COVID-19, Civil-Military Relations

## Abstract

The COVID-19 pandemic is giving way to increases in military engagements in health-related activities at the domestic level. This article situates these engagements amid issues of continuity, change, and resistance in contemporary redefinitions of military health roles. It positions the COVID-19 pandemic as a pivotal moment in global health military practice. I identify three emerging trends within national military responses to COVID-19: (1) Minimal technical military support; (2) Blended civil-military responses; and (3) Military-led responses. The dynamics that underpin each type of military involvement follow context-specific military political legacies. These levels of involvement also relate to national public health approaches and the degree of capacity within health care systems. Each identified trend points towards specific trajectories for the future co-constitution of global and local civil-military engagements.

## Introduction

The months that followed the WHO's declaration of the novel coronavirus outbreak as a Public Health Emergency of International Concern saw most states mobilising some level of military capacity. Militaries took on a wide variety of roles amid national responses. These ranged from setting up field hospitals in Serbia, Russia, or France, to delivering protective equipment or enforcing lockdowns in South Africa, Spain, or Italy. In some settings, like the Philippines or Indonesia, the military led the entire response. This article situates these COVID-19 military involvements amid the contemporary use of militaries in global health. It highlights issues of continuity, change, and resistance in military health-related roles. The article positions the pandemic as a pivotal event in global health military engagements. I identify three emerging trends in national military responses to COVID-19: (1) Minimal technical military support; (2) Blended civil-military responses; and (3) Military-led responses. Each of these trends provides a scale of military encroachment into national health apparatuses (see Table 1). They also point towards specific lacunae within health and political systems. Overarching dynamics characterise these involvements. These partake to a country's historical military legacy, the robustness of its civilian health system, and its public health approach (including pandemic preparedness models and delivery frameworks). Fomenting new COVID-19-related civil-military assemblages, these involvements will inescapably influence future local and global civil-military relations.

**Table 1.** Three trends in COVID-19 civil-military involvements.

	Trend types	Key characteristics	Response examples*
**1**	Minimal technical military support	Civilian leadership-military niche tasks in transportation and supply chain, border control	Japan, Taiwan, Canada, Kerala, Sweden, New Zealand, South Korea
**2**	Blended civil-military response	Civilian leadership-military support in organisation and logistics; air repatriations, border controls, mobile testing, quarantine and lockdown enforcement, emergency field hospitals	Nigeria, Kenya, US, France, UK, China, Vietnam, South Africa, Singapore
**3**	Military-led response	Military leadership in response planning and coordination, emergency hospitals, contact-tracing, surveillance, border controls, quarantine and lockdown enforcement	Indonesia, Philippines, Iran, Pakistan, Brazil, Peru

*Note*: These settings have exhibited the above key characteristics in the first six months of the coronavirus pandemic.

## Militaries as health actors

Often thought of as a last resort, militaries have become a preferred response in humanitarian crises, health emergencies, and pandemic preparedness.^1^ This presence has taken hold through the reciprocal increase of health activities within defence policy and that of militaries in the global health policy realm. This two-way process is owed to the interdependence of international and local civil-military health engagements. I understand global health military engagement as an understudied phenomenon linking foreign and domestic military health practices. This phenomenon has long institutional roots; militaries have historically used health activities to legitimise their presence in domestic and foreign settings.^2^ A global politics of medicine (linking medicine and warfare) can be traced back to colonial times^3^ and context-specific martial politics have carried through civilian institutions.^4^ Enduring French military cooperation (through the Pasteur Institutes’ worldwide presence), for example, bears witness to this legacy.^5^ Militaries have long contributed to medical innovation and population-level disease control efforts.^6^ The United States (US) Military Committee on Medical Research's development of anti-malarial chloroquine treatments during the Second World War^7^ or Major Walter Reed's yellow fever human experimentation programme^8^ are paradigmatic instances. COVID-19-related military engagements have, therefore, emerged amid a historical continuum linking health and military actors. This historical continuum is exacerbated by contemporary dynamics at both international and national levels.

At the national level, militaries usually encompass medical services. These services typically make up a small fraction of overall defence expenditures. The extent to which these entities and broader military resources are involved in national public health systems varies. Some militaries have no involvement in civilian health, while some participate in direct health care provision or public health efforts (for example, routine outreach health programmes or rural vaccination campaigns). In low- and middle-income countries (LMICs), militaries often fill the gaps in under-resourced health systems. These extensive health-related roles in particular have remained widely understudied. In most settings, military actors are involved in emergency response, preparedness, and planning. State level preparedness models will apply governmental strategies to maintain order and citizen welfare in emergencies.^9^ Entities like the military, non-governmental organisations (NGOs) or local authorities will assume various leadership roles amid these mechanisms (for example, early warning systems, scenario-based exercises, crisis communications, and stockpiling of essential supplies).^10^ National responses to COVID-19 are, in part, determined by preparedness models involving the military (such as disease simulation exercises)^11^ as well as the everyday influence of militaries in health care delivery and strategy.

• At the international level, contemporary strategic agendas have deepened and diversified military global health mandates.^12^ The language of stabilisation, capacity building, peacebuilding, and state-building has reinforced the link between defence and health objectives.^13^ Championed within Anglo-American defence policies, medical stability operations have become key areas in counterterrorism and counterinsurgency operations.^14^ These involvements often have internal ethical codes of conduct and means of engagement outside of traditional humanitarian frameworks.^15^ Traditional guidelines and mechanisms for civil-military cooperation and coordination^16^ are upheld to various extents in large-scale natural disasters and humanitarian emergencies. These types of operations host the most documented civil-military interactions.^17^ In peacetime, military engagements involve research and development, capacity building and partnerships, while^18^ bilateral health-related exercises^19^ are designed to uphold allied military capacity.^20^ Global health missions are often framed as win-wins; improving the health of foreign populations, while supporting homeland military strategy and capacity (for example, in homeland disease outbreaks, natural disasters or terrorist attacks).^21^ COVID-19 military responses are arising amid ongoing local and international military practices involving national, multilateral, bilateral, and regional forces.

Military engagements in global health have also been associated with the rise of the global health security paradigm. From the early 2000s, global health security became a prominent frame in global health policy. Proponents of global health security were initially concerned with increasingly globalised infectious diseases threats^22^ and the weaponisation of new pathogens.^23^ In the US, notably, a long tradition has linked national security and public health.^24^ There, biodefence advocates have actively framed disease in terms of national security since the 1990s.^25^ At the international level, various alliances further consolidated the health-security link.^26^ The year 2000 marked a historical shift when for the first time the UN Security Council deemed HIV/AIDS a threat to international peace and security. The impact of the HIV/AIDS epidemic on military personnel also formalised the health-security link.^27^ If framing a disease as a security threat has often led to its prioritisation on the international stage,^28^ it also tended to further security actor involvements in the health realm.^29^ Proponents of military engagement in public health see coherence in aligning security mandates with wider societal goals.^30^ This standpoint sees the inclusion of the military in wider health sector capability as a more efficient and holistic take on state capacity (that is, less silos, more synergy).^31^ Yet the safeguarding of populations against infectious diseases through security policies carries significant tensions (conflicting values).^32^ These partake to national security, transnational contagion, containment efforts (like social distancing). Tensions have emerged within global health security as a field of practice regarding *who* provides security and *what* constitutes a security threat.^33^ People-centered, human security, or rights-based perspectives have offered alternatives to traditional conceptions of security in the health realm.^34^ Amid the pandemic, governmental responses are guided by these differing (securitised-biomedical or people centred) approaches to public health.^35^ If traditional biosecurity approaches usually welcome military involvements, community-focused public health approaches tend to caution against them.^36^

Resistance to military involvements in health relates to specific areas of concerns. These partake to the adverse effects of politicising health interventions^37^ (for example, the manipulation of health goals for strategic outcomes) deemed unethical and counterproductive.^38^ The lack of respect of the Geneva conventions by militaries (growing civilian casualties, targeting of civilian institutions)^39^ and the adoption of health-related ‘hearts and minds’ type tactics has led to cognitive dissonance in international-level civil-military relations. Public health goals and humanitarian principles (for example, neutrality, impartiality, and independence) are often hard to reconcile with military mandates^40^ and institutional cultures.^41^ The conflation between civil and military roles is often deemed detrimental to delivery outcomes. Military health programmes (in disease surveillance, for instance) are thought more likely to be subject to geopolitical tensions and community suspicion.^42^ This distrust in turn risks having a knock-on effect on wider civilian health structures.^43^ Critics fear military involvement can be detrimental to advocacy initiatives, undermine primary care efforts or deter attention from the systemic root causes of ill health.^44^ Another apprehension lies in the potential slow takeover of civilian issues and institutions by militaristic culture and processes.^45^ The ensuing angst lies in military and intelligence organisations using their health mandates and authority to impede on civil liberties.^46^ These risks have led to the idea that civilians simply do better than the armed forces in global health emergency contexts.^47^ Here, military comparative advantage is relegated to limited technical activities (for example infrastructure, airlifts, airports, transport helicopters).^48^ If states have turned to their militaries for assistance in COVID-19, they also lack understanding of what that assistance can or should look like to establish parameters and limits of involvement.

These considerations are emerging amid a murky arena. We know very little about the outcome of military engagements in practice. Militaries increasingly prioritise global health engagements while failing to gather (or publish) evidence of whether they do in fact legitimise their presence or advance specific health targets.^49^ On the civilian side, scholarship has remained overwhelmingly concerned with military motives of engagements. Comprehensive programme reviews and health specific enquiries are extremely rare. Important, albeit limited, works have reviewed deployments during Zika,^50^ Influenza,^51^ and Ebola^52^ outbreaks. A lack of base-line data and understanding into pre-COVID-19 military health engagements undermines the potential for comparisons across regions or political systems. Anecdotal evidence, for instance, points to African militaries as frontrunners in military public health engagement.^53^ Yet these actors have remained largely overlooked. This lack of insight is inherent to the practical difficulty of conducting civil-military research. If some civil-military disease-research partnerships have long been institutionalised,^54^ limited forums connect academic, military, and health actors. When these collaborations do occur,^55^ they tend to be fomented amid antagonistic research goals. From the civilian side, most of these collaborations come about as a response to the potential detrimental effects of civil-military interactions; for example, risk management in civil-military relations or to avoid violence against health care workers.^56^ On the military side, civil-military research partnerships tend to give grounds for defence agendas.^57^ Militaries also often have a vested interest in data being classified. This dissimilitude in ethos and intentions has historically made collaborations difficult. These research challenges, combined with the constant recalibration and volatile nature of COVID-19 contagion trends, make for a complex research arena.

Despite our lack of understanding, pivotal events (such as humanitarian crises, epidemics, wars and now COVID-19) further entrench militaries as common actors in the health realm. These events produce shifts and leaps amid the historical continuum linking militaries and health practices. The 2014–16 West African Ebola epidemic remains a seminal example of this process. Militaries from France, Germany, the United Kingdom, China, Canada, and the United States were deployed on various support tasks amid this specific response. Foreign military deployments were put forward as the only answer to a capacity gap at international level.^58^ This contributed to raising the profile of global health activities within the armed forces involved.^59^ Seen as the primordial factor overturning the epidemic, these deployments consolidated health security practices outside of traditional humanitarian frameworks.^60^ They also provided capacity building towards COVID-19 responses,^61^ which are, in turn, fomenting new military practices within global health response mechanisms. These country-specific civil-military pathways will influence our collective perception of militaries as health actors. The coronavirus pandemic consequently stands as pivotal event in global civil-military relations. It brings urgency to establishing common definitions and frames of reference to apprehend health-related military engagements in all their complexity.

## Trend 1. Minimal technical military support

The first trend identified during the COVID-19 pandemic's initial stages (first six months) involved minimal military targeted technical assistance. This trend emerged amid deliberately civilian-led and operationalised responses. If they did not exhibit military involvements in the first months of the pandemic, these examples might be host to second stage military-related preparedness plans. However, these responses have intentionally limited military involvements to niche technical tasks in support of the civilian response. In these settings, specialised and sporadic military involvement often remained unused in the first months of the pandemic. This targeted military presence was, for instance, rolled out as part of the response in Japan. The country, which has a tight system ensuring civilian control of its military, has deployed its Self-Defense Forces (SDF) in low-key peripheral tasks, such as assisting the quarantine of arrivals at main airports.^62^ South Korea and Taiwan are also clear instances, despite both these settings having recent histories of military rule (up until 1980s).^63^ New Zealand, Sweden, or Canada also exhibited minimal technical military involvements. The common thread in these types of involvement lies in the mobilisation of highly technical military expertise (mainly logistical and transport competencies or boarder control). These niche components are deployed following contagion levels and related pressure on the civilian response capacity. In Canada, for instance, the prime minister made clear he steered away from military involvements in press conferences. Yet the Canadian province of Quebec deployed the Canadian military (trained by the Quebec section of the Canadian Red Cross) in care homes.^64^ These peripheral involvements are, therefore, giving way to new civil-society-public sector-military partnerships. Rare are countries where there has not been some form of involvement, if only symbolic. In Sweden, for example, a military crisis hierarchy management system borrowed from the North Atlantic Treaty Organization (NATO) was adopted to handle the influx of patients in Stockholm's main hospital.^65^ Yet some settings have had no military involvements. Costa Rica (which has no military) has remained an example of community compliance in the first six months of the pandemic. The country managed to stay at the low end of contagion rates compared to other Latin American settings during the first months of the pandemic (see Trend 2 and 3). Another example is the Indian state of Kerala. Its cheap and creative socioeconomic and logistical measures were met with praised results and did not require any form of military involvement.^66^ Kerala's legacy of primary health care service investment (adequately trained personnel, centralised management with both urban and rural institutional reach) and proactive leadership proved a winning recipe in these first months.^67^ State responses that exhibit minimal military engagements also showed better outcomes in the management of the disease and implementation of measures in these early months. What seems to be a common denominator is the reach of primary care capacity, adequate civilian pandemic preparedness and trust in public institutions.

## Trend 2. Blended civil-military responses

Blended civil-military responses make up the most common and broader mode of operation in domestic responses to COVID-19. From the pandemic's onset, China's People Liberation Army played a central role in the national response, setting the tone for subsequent responses.^68^ Blended civil-military responses are usually put forward to boost public health systems capacity. Often, they are rolled out to prevent systems from collapsing (through population control measures, for instance). Amid blended responses, national militaries take part in population-facing activities in parallel to, or embedded within, the civilian-led response.^69^ The latter will strive to harness the involvement of actors across ministries (be it defence, interior, health, transport, etc.) and civil society organisations.^70^ In these contexts, military support remains subordinate to the civilian response leadership in an effort of coordination and cooperation. Military logistics and transport capabilities are deployed to support procurement and distribution (of PPE or other medical equipment). Technical assistance such as medical air ambulance services, oxygen tankers transportation, aircrafts repatriations, research and laboratory capacity, mobile testing units or patient screening centres are also rolled out.^71^ Civil-military hospital capacity is a central component of this trend. The United Kingdom's NHS Nightingale Hospitals, Serbia's Belgrade Rair makeshift hospital, or the French field hospital units in Mulhouse are examples of joint military outlets rolled out to ease the pressure on civilian institutions.

If some regional commonalities are identified, countries will also exhibit stark differences in subnational experiences of civil-military engagements. This is the case in the United States where the military was dispatched at local level (for example, the US hospital ships dispatched in cities of New York and Los Angeles).^72^ In many settings, the armed forces are deployed to patrol streets in lockdown, disinfect public spaces, and support border control in attempts to halt transmission routes. Asian militaries with previous experience in Severe Acute Respiratory Syndrome (SARS) outbreaks were quickly deployed to respond to these specific tasks. The Singapore Armed Forces (a majority conscript force), for instance, was mobilised to distribute masks to the public, carry out contact tracing, and assist in medical screening at airports.^73^ Vietnam's robust state security apparatus was also deployed to counter the virus, taking part in the country's celebrated response. Vietnamese efforts to trace the contacts of infected travellers drew on personnel from the civil service, health workers, and the army,^74^ consolidating new civil-society-military partnerships.

Features and characteristics of blended involvements are also wide ranging. Military enforcement of pandemic measures has taken hold in LMICs where dense urban populations rely on subsistence economies. In these settings, militaries enforced lockdowns through various coercive measures. These draconian law enforcement or abuses are unfolding in cities of India,^75^ Nigeria, or Kenya.^76^ On the one hand, COVID-19 blended responses are building capacity at systems level, conveying more collaboration within statewide apparatuses. This can be achieved by fostering military triage capacity in treatment centres or allowing for the review of military medical services reach and mandates. This is especially the case in settings where the defence sector is already active in public health delivery practices. On the other hand, new arrays of tasks have also stretched the capacity of some underfunded and unprepared armed forces. Some military leaders have made clear (in the US, for example) that they saw the forces as unfit for countrywide health work.^77^ The South African National Defence Force, for instance, was deployed to combat COVID-19 (protect quarantine sites, provide health support services, deliver food, help police in containment efforts, conduct roadblocks). During this period, they also remained in action amid peacekeeping missions and other security-related deployments at home and overseas.^78^ A lack of capacity at health system level, a lack of trust in public institutions and a lack of training is likely to undermine military involvements within blended civil-military responses.

## Trend 3. Military-led responses

In some settings, the military has taken the leadership of the entire COVID-19 response. These primarily militarised responses are unfolding in Indonesia, Sri Lanka,^79^ Myanmar, Thailand, and the Philippines. Militaries in Argentina, Brazil, Mexico, Chile and Uruguay, Ecuador and Peru, are all displaying some levels of blended civil-military responses quickly shifting towards military leadership.^80^ In Brazil, for instance, the ministry of health leadership has gradually passed into military hands amid the worsening of the crisis.^81^ Some local-regional responses shifted to military control when deemed unmanageable for civilian systems and leadership. In Ecuador, for instance, the province surrounding Guayaquil has been placed under military jurisdiction.^82^ Military is leadership of COVID-19 responses will have political repercussions in settings where are likely to reverse harshly acquired civilian control of institutions. There they are met with predictable pushbacks from civil society groups. This is the case in Indonesia and the Philippines where fears that the military is trying to ‘clawback civilian power’ are emerging amid COVID-19 response leadership.^83^ Shifts in the balance of power of already hybrid political systems or systems with heavy military influence are intensifying amid COVID-19 responses. This is the case in Iran^84^ and in Pakistan,^85^ which are waging controversial responses amid enduring challenges in civil-military relations. In these settings, the pandemic further encroaches military presence into domestic civilian affairs. This is particularly worrying in settings where the military leads responses amid disenfranchised minority groups, like in the Sri Lankan North-East Tamil region^86^

Military takeover of traditionally civilian roles point to an often neglected dynamic in civil-military enquiries; if there is obviously a recourse to the military when civilian capacity fails (vacuum filling pull factor), there is also a push factor associated with the military itself and its willingness to undertake health duties. Here, militaries are not necessarily invoked but also position themselves as responders capable of delivering required services. This push factor in undertaking civilian roles amid the crisis and encroaching on state institutions can be internal to defence institutions and not necessarily marshalled through centralised decision-making. It will be crucial to monitor whether emergency military powers are transferred back to civilian authorities in the contexts mentioned above.^87^

## Civil-military pathways

If comparing governmental strategies has offered some learning points amid the pandemic, it has also fuelled misinformation.^88^ A multitude of confounding factors underpin types of governmental responses. These include differing political systems, levels of institutional capacity and political legitimacy, justice systems, media freedom, and reliability. Extremely context-specific national and regional experiences will constitute new sets of norms and practices linking health and defence institutions. Levels of military involvement in health also vary according to contagion-levels, political climates, and institutional legacies. Other factors such as the density of the population or the competency of state leaders^89^ and health ministers^90^ will also influence the recourse to military responses. In some settings, political leadership's acceptance of the virus (that is, whether leaders believed coronavirus was real) was a significant factor for military involvements. In Burundi^91^, Brazil,^92^ and the US,^93^ COVID-19 presidential denial has led to delayed measures. These delays have allowed for significant rises in contagion levels, which, in turn, led to further military involvements. The accrued presence of non-military security actors adds to the difficulty of discerning causes and extent of militarised involvements. Militarised police, immigration border agents, or private military companies were documented having frontline roles in local responses.^94^ Non-State Armed Groups who have often used health interventions to establish legitimacy^95^ and criminal gangs have capitalised on COVID-19 to tighten population control, sometimes providing services amid the pandemic.^96^ These types of involvements can go unnoticed amid traditional civil-military enquiries. Differing contextual realities fuel cross-case variability and impact on our ability to draw conclusions from military trends of involvement.

Despite these challenges in case variability, the coronavirus pandemic stands as an unprecedented opportunity to evaluate civil-military work and policy. The breath of response types offers insights into military motives of engagement and their added value and limitations. These occurrences will also refine our understanding of governmental biosecurity preparedness decision-making processes and mechanisms. At the same time, the social determinants and institutional capacity gaps fuelling this pandemic should not be subordinated to discussions surrounding the use of security and military capacity. Civil-military scholarship in global health has traditionally been focused on best practices for militaries (that is, how-to best use militaries in the civilian realm). If these are important questions, the ways in which militaries become involved in COVID-19 point towards more pressing considerations. Trends of engagements clearly show that military capability is overwhelmingly used to compensate institutional lacunae in the civilian realm. A key question therefore lies in how to boost civilian capacity at delivery level (for instance in technical support, community contact tracing or treatment). Systematically reviewing military engagements in COVID-19 will provide invaluable insights into civilian capacity gaps. In this way, military involvements become parameters for civilian institutional capacity helping build stronger public health systems and more reactive social responses.

Equally important is the community impact and perception of these interventions, and whether military engagements make people feel safe. Community acceptance of these interventions is widely under studied and appears absent from decision-making processes. Gender is a paramount example. We know that the socioeconomic impacts of pandemics affect women disproportionately through risk of domestic violence, closure of sexual and reproductive care services, and economic loss.^97^ We also know that (especially in conflict and postconflict contexts) securitised discourse linking disease and militaries can play into gender-based vulnerabilities.^98^ COVID-19 responses and recovery efforts will need to be tailored to support women; the impact of military engagements should not be neglected amid these efforts. In many ways, the social determinants of the pandemic outcomes for vulnerable population or ethnic minorities^99^ cautions against militarised responses. Regionally focused military engagements have been linked to targeted population abuses. This was the case in the Indian part of Kashmir^100^ or in the occupied West Bank,^101^ for example. Critical attention should be put on barriers to accessing military-provided services as well as on coercive health-related engagements in vulnerable communities.

The type of approach to COVID-19 (biomedical, securitised, or community-led) will inherently make up the scale of military involvement. A lack of adapted strategy and discourse (such as regional technical guidance) for COVID responses in low-income countries (LICs) have led to monolithic lockdown-type strategies in the first months of the pandemic.^102^ These types of strategies have often added fuel to the fire in settings where people rely on daily subsistence and in crowded slum areas.^103^ Biomedical securitised COVID-19 national responses (involving military deployments, heavy lockdown measures, and border closures) appear in stark opposition to preventive strategies that prioritise community social acceptability and supportive public health services.^104^ In many LICs, the diversion of resources away from COVID-19 preventive measures (for example, imposing quarantines as opposed to shielding) could mean non-locally adapted biomedical models of intervention.^105^ A protracted pandemic, which exacerbates socioeconomic inequalities, calls for sustained cooperation, communication, and participatory decision-making.^106^ Enquiries into *whether* or *how* these requirements can be reconciled with military involvements are pressing.

## Emerging trends and collective perceptions

Framing the pandemic as a security threat influences policy and practice linking health and military realms amid all types of responses. The wide-reaching socioeconomic effects of COVID-19 and associated emerging hybrid security threats (for example, so-called infodemics and targeted cyber-attacks on research and web entities)^107^ are leading to new civilian-military response mechanisms. The North Atlantic Treaty Organisation (NATO), for instance, advocates for its members’ societal resilience to invisible and hybrid threats amid COVID-19.^108^ This comprehensive non-military-centric preparedness approach means governments, militaries, businesses, and civil society work together against emerging threats (such as disinformation campaigns).^109^ Governments, in task shifting control and treatment measures between civilian public institutions, the military, and community groups (for example, in care homes or for vaccine roll outs) are also creating new civil-society securitised assemblages.^110^ This incorporation of the life sciences and public health into the national security apparatuses is not new.^111^ But it is exacerbated in COVID-19 as security problems and civilian capabilities gaps are merged in militarised language. The pandemic has fostered examples of think tank publications praising the transferability of military operational culture for civilian institutions.^112^ These types of discursive acknowledgements of civil-military transferability (through the language of hierarchy, efficiency, and leadership) further normalise the health-military association. Military and war metaphors in the public discourse relating to COVID-19 (for example, ‘invisible enemy’, ‘frontline’, ‘duty’) reinforce statist thinking and state power.^113^ These metaphors can risk closing off alternative ways of understanding the disease and what fuels it (for example, the social determinants making populations vulnerable).^114^ These rapprochements (between health and military sectors) take hold through indirect pathways of language and practices amid civilian entities across all response trends. Until recently on the fringe of global health scholarship and practice, COVID-19 could normalise the military-health link, making it more palatable in the public domain.

Military experiences in COVID-19 responses will have geopolitical implications. Military actors’ catch-22 lies in the ability to maintain their primary functions of war and deterrence in the midst of internal pandemic pressures.^115^ Known carriers and vectors of infectious diseases, militaries will seek to prioritise their own personnel's health and operational readiness.^116^ If the pandemic keeps militaries busy, hence moderating risks of external confrontation, it is also altering the way they operate, perceive themselves and engage with each other.^117^ Examples of COVID-19 military diplomacy have already taken place. Russian military personnel deployed, for instance in the North of Italy,^118^ while the Pakistani military donated PPE to the US Army.^119^ Pending military biomedical innovation (in the form of cures or vaccines) also give the impetus for military presence in the health realm. US Operation Warp Speed, which used military research facilities for vaccine development, is one example of such involvements.^120^ The advent of the different vaccines might mean further mobilisation of defence institutions to protect stockpiles, enhance laboratory, or supply chain capacity. Grand-scale vaccination rollouts, which necessitate clear chains of command and coordination, make military expertise a go-to in many settings (even if some capacity can exist within civilian organisations). For critics, these involvements are likely to equate to further protectionism, border closures, geopolitical tensions, mistrust, or confrontations. Conversely, proponents of inclusive military involvements will see better coordination and efficiency across state apparatuses. Faced with pandemic-induced economic crisis disrupting military spending,^121^ defence leadership might choose to revaluate domestic roles. Faced with financial constraints and an enhanced portfolio of activity, positioning the armed forces as population-based health delivery actors could help justify defence funding and expenditure. Amid ideological privatisation and austerity measures undermining public institutional health capacity, militaries could become the alternative institutional response mechanism. These potential changes pose fundamental questions for future civil and military health roles (and eventually for cosmopolitanism as military practice).

Domestically, COVID-19 military engagements are drawing up new internal ethical frameworks and doctrines. These types of military-internal operational frameworks have, over the past decade, increasingly been put forward as alternatives to traditional humanitarian guidelines in global health military contexts.^122^ Amid this pandemic, national emergency-related laws have conferred governments further access to martial power, sometimes risking undermining hard-acquired civil liberties.^123^ Against a background of fast developing surveillance practices,^124^ issues of technological control and authoritarianism have raised the world around.^125^ In some contexts, the coercive nature of local armed forces and the slippery slope nature of authoritarian measures have led to human rights abuses in the name of public health.^126^ New legal and ethical frameworks and instruments relating to COVID-19 measures (for example, ethical medical prioritisation, tracing, surveillance, quarantine) will need to balance human rights protection and inclusive public health promotion.^127^ These could in turn lead to accrued civilian control of military practices, or to increased military control of civilian affairs. These new local civil-military frameworks and practices will impact on future international-level civil-military coordination and cooperation.

## Conclusion

The coronavirus pandemic stands as a pivotal moment in the contemporary presence of militaries in global health. As confinement measures were enforced and health systems were put under strain, military deployments have unfolded through three clear trends of engagement: (1) Minimal technical military support; (2) Blended civil-military responses; and (3) Military-led responses. In light of these three levels of participation, it appears that the recourse to military is threefold. First, it follows a country's historical legacy in civil-military relations and perception of military delivery. Various historical and political trajectories led to the institutionalisation of military public health work and subsequent COVID-19 responses. Second, these involvements occur to fill gaps when health systems are overwhelmed. This is universal, follows contagion threat levels and health systems’ ability to cope with the epidemic pressure. This is also a gradual process, more widespread in states with weaker health systems or where the military has historically run civilian-serving services. A third and important dynamic is compounded by top-down pandemic preparedness and delivery frameworks. When adopting securitised biomedical responses, countries with weak health systems need to recourse to top-down (often military) means. In COVID-19, these responses are marshalled through the military to enforce measures such as lockdown, surveillance, border closures, or contact tracing. The ability to command, through military means, remains a double-edged sword. It allows for stringent responses but threatens citizenship rights and community trust so crucial in epidemics. The direct and indirect involvements of the military in COVID-19 national responses have led to increases in policy and practice linking military and health actors. This is likely to have a normative impact, further entrenching militaries as common actors in the health realm. Global health and IR scholarship should focus on the ways in which civilian institutional lacunae are compensated through military means. These insights will allow for better societal resilience amid the pandemic and future emergency responses. They will also provide empirical evidence to the wider questions of both *if* and *how* militaries have a role in global health.

## Footnotes

1 Joshua Michaud , ‘Militaries and global health: Peace, conflict, and disaster response’, The Lancet, 393 (2019), pp. 276–8610.1016/S0140-6736(18)32838-130663597.

2 See, for instance, Coreen M. Beaumier , ‘United States military tropical medicine: Extraordinary legacy, uncertain future’, PLoS Neglected Tropical Diseases, 7:12 (2013), pp. 1–610.1371/journal.pntd.0002448PMC387325824386494.

3 Alison Howell, ‘The global politics of medicine: Beyond global health, against securitisation theory’, Review of International Studies, 40:5 (2014), pp. 961–87.

4 Alison Howell, ‘Forget “militarization”: Race, disability and the “martial politics” of the police and of the university’, International Feminist Journal of Politics, 20:2 (2018), pp. 117–36.

5 See Anne Marie Moulin, ‘Patriarchal science: The network of the overseas pasteur institutes’, in Patrick Petitjean, Catherine Jami, and Anne Marie Moulin (eds), Science and Empires, Boston Studies in the Philosophy of Science, Vol. 136 (Springer: Dordrecht, 1992), pp. 307–22; see also Liora Bigon, ‘A history of urban planning and infectious diseases: Colonial Senegal in the early twentieth century’, *Urban Studies Research* (2012), pp. 1–12.

6 See, for instance, Randall M. Packard, A History of Global Health: Interventions Into the Lives of Other People (Baltimore: The Johns Hopkins University Press, 2016), p. 432; see also Roberto Zaugg, ‘Guerre, maladie, empire: Les services de santé militaires en situation coloniale pendant le long XIX^e^ siècle’, Histoire, médecine et santé, 100 (2016), pp. 9–16.

7 Sophie Harman, Global Health Governance (Abingdon: Routledge, 2012), p. 93.

8 See, for instance, Geoffrey Quail, ‘The debt tropical medicine owes to the military’, Journal of Military and Veterans’ Health, 23:3 (2015), pp. 18–21.

9 Andrew Lakoff, Unprepared: Global Health in a Time of Emergency (Oakland: University of California Press, 2017), p. 20.

10 Ibid.

11 Most notably Operation Dark Winter lessons for COVID-19; see Mark Perry, ‘America's pandemic war games don't end well’, *Foreign Policy*, available at: {https://foreignpolicy.com/2020/04/01/coronavirus-pandemic-war-games-simulation-dark-winter/} accessed 30 October 2020.

12 See Colin McInnes and Simon Rushton, ‘Health for health's sake, winning for God's sake: US global health diplomacy and smart power in Iraq and Afghanistan’, Review of International Studies, 40:5 (2014), pp. 835–57.

13 See, for instance, Jean-Paul Chretien, ‘US military global health engagement since 9/11: Seeking stability through health’, Global Health Governance, IV:2 (2011), pp. 1–12; Derek Licina, ‘The military sector's role in global health: Historical context and future direction’, Global Health, 6:1 (2012), pp. 1–30.

14 See, for instance, McInnes and Rushton ‘Health for health's sake’.

15 Thomas Falconer Hall, Simon Horne, and D. Ross, ‘Comparison between defence healthcare engagement and humanitarian assistance’, BMJ Military Health (2020), pp. 1–33221768710.1136/bmjmilitary-2020-001437PMC9685730.

16 Namely the Oslo Guidelines: ‘Guidelines on the Use of Foreign Military and Civil Defence Assets in Disaster Relief’, Office for the Coordination of Humanitarian Affairs (2007); International Humanitarian Law prerogatives; or international NGOs’ internal guidelines.

17 See, for instance, Shao Xiaohong, ‘The role of health sectors in disaster preparedness: Floods in southeastern China, 1991’, Prehospital and Disaster Medicine, 8:2 (1993), pp. 173–510155464; Wiley C. Thompson, ‘Success in Kashmir: A positive trend in civil-military integration during humanitarian assistance operations’, Disasters, 34:1 (2010), pp. 1–151945990210.1111/j.1467-7717.2009.01111.x; Jennifer Leaning and Debarati Guha-Sapir, ‘Natural disasters, armed conflict, and public health’, The New England Journal of Medicine, 26:2 (2014), pp. 147–5410.1056/NEJMc131550724552339.

18 Michaud et al., ‘Militaries and global health’, p. 277.

19 These are common practice but have received minimal scholarly attention, for example, the ‘Exercise Khan Quest’, an annual civil-military peacekeeping exercise (involving forty countries) co-hosted by the Mongolian Armed Forces and the US Department of Defence, see Michaud et al., ‘Militaries and global health’, p. 278.

20 See Jean-Paul Chretien , ‘The importance of militaries from developing countries in global infectious disease surveillance’, Bulletin of the World Health Organization, Vol. 10 (2007), pp. 1–1210.2471/BLT.06.037101PMC263623517486207; see also James Alan Balcius, ‘Maritime Military Humanitarian Civic Assistance Missions: Resource Use, Coordination, and Governance to Improve Global Health’ (peer review thesis, University of California San Diego, 2017); Adam C. Levine and David P. Polatty IV, US Naval War College, ‘Civilian-Military Humanitarian Response Workshop Summary Report’ (2018), available at: {https://digital-commons.usnwc.edu/workshop-reports/1} accessed 29 December 2020.

21 Military-affiliated authors tend to link foreign global health engagements to homeland protection purposes; see Matt Pueschel, ‘Global medical mission training: The Department of Defense is making headway in training for global medical missions’, Journal of Special Operations Medicine, 11:4 (2011), pp. 15–192211372110.55460/U0WR-0J5E; Sheena M. Eagan, ‘Global health diplomacy and humanitarian assistance: Understanding the intentional divide between military and non-military actors’, *Journal of the Royal Army Medical Corps* (2018), pp. 244–7; Heather C. King , ‘Shipboard global health engagement missions: Essential lessons for military healthcare personnel’, Military Medicine, 0/0:1 (2019), pp. 1–710.1093/milmed/usz11331141136.

22 See, for example, Colin McInnes and Kelly Lee, ‘Health, security and foreign policy’, Review of International Studies, 32:1 (2006), pp. 5–23; see also Colin McInnes and Anne Roemer-Mahler, ‘From security to risk: Reframing global health threats’, International Affairs, 93:6 (2017), pp. 1313–37. For a contemporary history of such interventions, see Andrew Lakoff and Stephen Collier (eds), Biosecurity Interventions: Global Health and Security in Question (New York: Columbia University Press, 2008), p. 308.

23 Stefan Elbe, ‘Should health professionals play the global health security card?’, The Lancet, Vol. 378 (2011), pp. 220–110.1016/S0140-6736(11)61114-8PMC713791921770062.

24 For US internal and global security efforts, see the National Intelligence Council's Assessments on Infectious Diseases; notably the ‘National Intelligence Estimate on the Global Infectious Disease Threat’ (2000); ‘The Next Wave of HIV/AIDS: Nigeria, Ethiopia, Russia, India, and China’ (2002); and ‘SARS: Down But Still a Threat (2003).

25 See, for instance, the integration of pandemic preparedness into the counterterrorism homeland security planning, in Lakoff, *Unprepared*, pp. 30–3, 50–5.

26 Among them the Global Health Security Initiative (2001) and The World Health Assembly resolution entitled ‘Global Health Security: Epidemic Alert and Response’ (2001).

27 Alex de Waal, Jennifer F. Klot, Manjari Mahajan et al., ‘HIV/AIDS, Security and Conflict: New Realities, New Responses AIDS’, Security and Conflict Initiative (ASCI) (2001).

28 Sara E. Davies, ‘Securitizing infectious disease’, International Affairs, 84:2 (2008), pp. 295–313.

29 This trade-off was first highlighted in Stephen Elbe's account of the securitisation of the HIV-AIDS pandemic; see Stefan Elbe, ‘Should HIV/AIDS be securitized? The ethical dilemmas of linking HIV/AIDS and security’, International Studies Quarterly, 50:1 (2006), pp. 119–44; Stefan Elbe, ‘Should health professionals play the global health security card?’, The Lancet, 378 (2011), pp. 220–110.1016/S0140-6736(11)61114-8PMC713791921770062; see also Clare Wenham, ‘The oversecuritization of global health: Changing the terms of debate’, International Affairs, 95:5 (2019), pp. 1093–110.

30 See, for example, Nicholas Thomson , ‘Harnessing synergies at the interface of public health and the security sector’, The Lancet, 393 (2019), p. 20910.1016/S0140-6736(18)32999-430663581; Frederick M. Burkle, ‘Throwing the baby out with the bathwater: Can the military's role in global health crises be redeemed?’, Prehospital and Disaster Medicine, 28:3 (2013), p. 19810.1017/S1049023X1300016223406945.

31 See James Alan Balcius, ‘Maritime Military Humanitarian Civic Assistance Missions: Resource Use, Coordination, and Governance to Improve Global Health’ (peer reviewed thesis dissertation, University of California San Diego, 2017); see also Eugene V. Bonventre and Valérie Denux, ‘Military health diplomacy’, in Thomas. E. Novotny, Ilona Kickbusch, and Micheala Told (eds), *21st Century Global Health Diplomacy* (Singapore: World Scientific, 2013), p. 207.

32 Christian Enemark first documented these trade-offs in *Biosecurity Dilemmas: Dreaded Diseases, Ethical Responses, and the Health of Nations* (Washington, DC: Georgetown University Press, 2016), pp. 1–226.

33 See, for instance, Simon Rushton, ‘Global health security: Security for whom? Security from what?’, *Political Studies*, 59:4 (2011), pp. 779–96.

34 See, for instance, Sara E. Davies, ‘What contribution can International Relations make to the evolving global health agenda?’, *International Affairs*, 86:5 (2010), pp. 1167–90.

35 Rene Loewenson, Kristen Accoe, Nitin Bajpai et al., ‘Reclaiming comprehensive public health’, *BMJ Global Health* (2020), pp. 1–6.

36 See William Aldis, ‘Health security as a public health concept: A critical analysis’, *Health Policy and Planning*, 23:6 (2008), pp. 369–75.

37 These apprehensions echo broader security-development nexus anxieties; see Mark Duffield, *Development, Security and Unending War: Governing the World of Peoples* (Cambridge: Polity Press, 2007); see also Micheal Young, ‘Development at gunpoint? Why civilians must reclaim stabilization aid’, *Foreign Affairs* (2010), available at: {https://www.foreignaffairs.com/articles/2010-12-19/development-gunpoint} accessed 28 June 2020.

38 Shoaib Fahad Hussain et al., ‘Eradicating polio in Pakistan: An analysis of the challenges and solutions to this security and health issue’, *Globalization and Health*, 12:1 (2016), pp. 1–9; Kathy Barker, Amy Hagopian, and James Pfeiffer, ‘Militaries and global health: Correspondence’, *The Lancet*, 394 (2019), p. 917.

39 Repeated attacks by national and coalition forces on hospitals in Syria, Yemen, and Afghanistan come as striking examples; see Preeti Patel et al., ‘Documenting attacks on health workers and facilities in armed conflicts’, *Bulletin of the World Health Organisation*, 95:1 (2017), pp. 79–81.

40 See, for example, Nicolas de Torrente, ‘Humanitarian NGOs must not ally with military’, *European Affairs*, 7 (2006), available at: {https://www.europeaninstitute.org/index.php/component/content/article/38-european-affairs/springsummer-2006/156-humanitarian-ngos-must-not-ally-with-military} accessed 29 July 2020; see also McInnes and Rushton, ‘Health for health's sake’; Ryoa Chung, ‘The securitization of health in the context of the war on terror: National security and global health: the conflict of imperatives’, *Medicine, Conflict and Survival*, 33:1 (2017), pp. 32–40; Jonathan Kennedy et al., ‘Militaries and global health: Correspondence’, *The Lancet*, 394 (2019), pp. 916–17.

41 For clashes in medical and military roles, see Stuart Gordon, ‘The military physician and contested medical humanitarianism: A dueling identity?’, *Social Science and Medicine*, 120 (2014), pp. 421–9; S. M. Eagan Chamberlin, ‘Medicine as a Non-Lethal Weapon: The Ethics of “Winning Hearts and Minds”’ (2015), available at: {http://www.ethikundmilitaer.de/en/full-issues/20151-medical-ethics/chamberlin-medicine-as-a-non-lethal-weapon-the-ethics-of-winning-hearts-and-minds/}accessed 24 July 2020; Kenneth W. Bernard, ‘Health and national security: A contemporary collision of cultures’, *Biosecurity and Bioterrorism* (2013).

42 This was the case in 2010 when an US Navy laboratory had to be closed down in Indonesia due to national sensitivities and suspicion; see Michaud et al., ‘Militaries in global health’, p. 278.

43 See, for example, Médecins Sans Frontières fear of conflation of roles and structures of care in Gordon, ‘The military physician and contested medical humanitarianism’; in conflict and postconflict settings, see Hakan Seckinelgin, Joseph Bigirumwami, and Jill Morris, ‘Securitization of HIV/AIDS in context: Gendered vulnerability in Burundi’, *Security Dialogue*, 41:5 (2010), pp. 515–35.

44 See, for instance, Adia Benton, ‘Whose security? Militarization and securitization during West Africa's Ebola outbreak’, in Sokhieng Au and Michiel Hofman (eds), *The Politics of Fear: Médecins sans Frontières and the West African Ebola Epidemic* (Oxford: Oxford University Press, 2017).

45 Elbe, ‘Should HIV/AIDS be securitized? The ethical dilemmas of linking HIV/AIDS and security’; Elbe, ‘Should health professionals play the global health security card?’.

46 Sandra S. Maclean, ‘Microbes, mad cows and militaries: Exploring the links between health and security’, *Security Dialogue*, 39:5 (2008), pp. 475–94; Benton, ‘Whose security?’.

47 de Waal, ‘Militarizing global health’, p. 8.

48 Ibid.

49 See McInnes and Ruston, ‘Health for health's sake’; also Maclean, ‘Microbes, mad cows and militaries’; for impact on case maternal and sexual health specifically, see also Laura Baringer and Steve Heitkamp, ‘Securitizing global health: A view from maternal health’, *Global Health Governance*, 4 (2011), pp. 1–21.

50 Clare Wenham and Deborah B. L. Farias, ‘Securitizing Zika: The case of Brazil’, *Security Dialogue*, 50:5 (2019), pp. 398–415.

51 Christopher Watterson and Adam Kamradt-Scott, ‘Fighting flu: Securitization and the military role in combating influenza’, *Armed Forces and Society*, 42:1 (2016), pp. 145–68.

52 Adam Kamradt-Scott et al., ‘Saving Lives: The Civil-Military Response to the 2014 Ebola Outbreak in West Africa’, University of Sydney (2015), available at: {http://sydney.edu.au/arts/ciss/downloads/SavingLivesPDF.pdf} accessed 1 May 2020.

53 Charles Raymond Dotou et al., ‘Toward strengthening the health politics in Africa: The military health system and its contribution to health policy in Senegal’, *Bulletin de la Societe de pathologie exotique*, 97 (2004).

54 Several military medical research institutions are designated WHO Collaborating Centres; see Michaud et al. ‘Militaries and global health’, p. 281.

55 See, for examples of collaboration, Josiah Kaplan, ‘Summary of the Oxford Humanitarian Innovation Project (HIP)-Royal United Services Institute (RUSI) Pandemic Civil-Military Coordination Workshop’ (2017), pp. 1–7; Samuel Boland, ‘The Next Ebola: Considering the Role of the Military in Future Epidemic Response’, Centre on Global Health Security Roundtable Summary, Chatham House (2017), pp. 1–10; Gemma Bowsher, Fawzia Gibson-Fall, and Rose Bernard, ‘Negotiating the Humanitarian Space: New Opportunities for Action and Research’, conference report, *Journal of the Royal Society of Medicine* (2018).

56 For example Kamradt-Scott et al., ‘Saving Lives’; Patel et al., ‘Documenting attacks on health’; Bowsher et al., ‘Negotiating the Humanitarian Space’.

57 See, for example, Roberto N. Nang and Keith Martin, ‘Global health diplomacy: A new strategic defense pillar’, *Military Medicine*, 18:1 (2017), pp. 1456–60.

58 Sophie Harman and Clare Wenham, ‘Governing Ebola: Between global health and medical humanitarianism’, *Globalizations*, 15:3 (2018), pp. 362–76.

59 See, for instance, Heather Draper and Simon Jenkins, ‘Ethical challenges experienced by UK military medical personnel deployed to Sierra Leone (operation GRITROCK) during the 2014–2015 Ebola outbreak: A qualitative study’, *BMC Medical Ethics*, 18:1 (2017), pp. 1–13; John Whitaker and D. Bowley, ‘Beyond bombs and bayonets: Defence engagement and the defence medical services’, *Journal of the Royal Army Medical Corps*, 165:3 (2019), pp. 140–2.

60 Wenham, ‘Oversecuritization of global health’.

61 See, for example, James Bashall, ‘COVID-19 & the Armed Forces: Lessons from Sierra Leone’, Defence IQ (2020), available at: {https://www.defenceiq.com/air-land-and-sea-defence-services/editorials/covid-19-the-armed-forces-lessons-from-sierra-leone} accessed 20 July 2020. See also Chris Gibson, ‘Ebola lessons can be used in COVID-19 fight, former army medical instructor says’, *Forces Net*, available at: {https://www.forces.net/news/health-and-fitness/ebola-lessons-can-be-used-covid-19-fight-former-army-medical} accessed 20 July 2020.

62 Euan Graham, ‘The Armed Forces and COVID-19’, International Institute for Strategic Studies (2020), available at: {https://www.iiss.org/blogs/analysis/2020/04/easia-armed-forces-and-covid-19} accessed 29 July 2020.

63 Ibid.

64 Stéphanie Marin, ‘CHSLD: une première cohorte formée par la Croix-Rouge déployée lundi’, *La Presse* (2020), available at: {https://www.lapresse.ca/covid-19/2020-07-01/chsld-une-premiere-cohorte-formee-par-la-croix-rouge-deployee-lundi.php} accessed 29 June 2020; see also Amine Esseghir, ‘La Croix-Rouge forme les militaries’, *Métro* (2020), available at: {https://journalmetro.com/local/ahuntsic-cartierville/2442251/la-croix-rouge-forme-les-militaires/} accessed 29 July 2020.

65 ‘Military protocol in Swedish hospitals prepare for worst outbreak’, *Aljazeera English* (2020), available at: {https://www.youtube.com/watch?v=9YYvyHcHkMc} accessed 29 June 2020.

66 ‘Vietnam and the Indian state of Kerala curbed Covid-19 on the cheap’, *The Economist* (2020), available at: {https://www.economist.com/asia/2020/05/09/vietnam-and-the-indian-state-of-kerala-curbed-covid-19-on-the-cheap} accessed 29 June 2020.

67 Oommen Kurian, ‘How the Indian state of Kerala flattened the coronavirus curve’, *The Guardian* (2020), available at: {https://www.theguardian.com/commentisfree/2020/apr/21/kerala-indian-state-flattened-coronavirus-curve} accessed 29 June 2020.

68 Graham, ‘The armed forces and COVID-19’.

69 Joseph Kazibwe et al., ‘Using Military Health Systems in the Response to COVID-19’, Centre for Global Development (2020), available at: {https://www.cgdev.org/blog/using-military-health-systems-response-covid-19} accessed 29 June 2020.

70 Martin Bricknell, ‘How Militaries Around the World Joined the Battle against COVID-19’, King's College London (2020), available at: {https://www.kcl.ac.uk/how-militaries-around-the-world-joined-the-battle-against-covid-19} accessed 29 June 2020.

71 Ibid.

72 Ross Barkan, ‘The vacant Comfort hospital ship is a symbol of our coronavirus failure’, *The Guardian* (2020), available at: {https://www.theguardian.com/commentisfree/2020/apr/03/comfort-ship-new-york-coronavirus-failure-symbol} accessed 1 July 2020

73 Vivian Ng, ‘How Singapore's military is fighting COVID-19, *The Diplomat* (2020), available at: {https://thediplomat.com/2020/03/how-singapores-military-is-fighting-covid-19/} accessed 29 June 2020.

74 ‘Vietnam and the Indian state of Kerala curbed Covid-19 on the cheap’, *The Economist*.

75 Violence Lab, ‘Pandemic and the State's Response: Understanding Lockdown Deaths in India’ (2020), available at: {https://thepolisproject.com/pandemic-and-the-states-response-understanding-lockdown-deaths-in-india/#.XvtV6y2w3OQ} accessed 29 June 2020

76 Olewe Dickens, ‘Coronavirus in Africa: Whipping, shooting and snooping’, *BBC* (2020), available at: {https://www.bbc.co.uk/news/world-africa-52214740} accessed 29 June 2020; News Wires, ‘Security forces use violent tactics to enforce Africa's coronavirus shutdowns’, *France 24* (2020), available at: {https://www.france24.com/en/20200401-security-forces-use-violent-tactics-to-enforce-africa-s-coronavirus-shutdowns} accessed 29 June 2020.

77 This lack of capacity, for instance, was highlighted in Pentagon interviews; see Jeff Shogol, ‘The US military is not the silver bullet in the fight against the coronavirus, Pentagon officials say’, *Task and Purpose* (2020), available at: {https://taskandpurpose.com/news/military-limited-response-coronavirus} accessed 29 July 2020; on reluctance because of detrimental aspects to democracy, see Kori Shake, ‘Send in the Marines? Not to Fight Coronavirus’, American Enterprise Institute (2020), available at: {https://www.aei.org/op-eds/send-in-the-marines-not-to-fight-coronavirus/} accessed 29 July 2020.

78 Lindy Heinecken, ‘COVID-19: South Africa's neglected military faces “mission impossible”’, *The Conversation* (2020), available at: {https://theconversation.com/covid-19-south-africas-neglected-military-faces-mission-impossible-133250} accessed 29 June 2020

79 Thusiyan Nandakumar, ‘A Military Mindset: Sri Lanka's Response to the Coronavirus Pandemic’, The Polis Project (2020), available at: {https://thepolisproject.com/militarization-of-medicine-sri-lankas-response-to-the-coronavirus-pandemic/#.XvtWpS2w3OT} accessed 29 June 2020

80 Kristina Mani, ‘“The soldier is here to defend you”: Latin America's militarized response to COVID-19’, *World Politics Review* (2020), available at: {https://www.worldpoliticsreview.com/articles/28700/from-peru-to-venezuela-military-forces-take-the-lead-in-coronavirus-responses} accessed 29 June 2020

81 Laura Gamba, ‘President Bolsonaro's military appointments often said to have very limited to no public health experience’, *AA* (2020), available at: {https://www.aa.com.tr/en/americas/bolsonaro-militarizes-healthcare/1910789} accessed 29 June 2020

82 Mani, ‘“The soldier is here to defend you”’.

83 Nyshka Chandran, ‘The pandemic has given armies in Southeast Asia a boost’, *Foreign Policy* (2020), available at: {https://foreignpolicy.com/2020/06/15/coronavirus-pandemic-army-military-southeast-asia-boost-indonesia-philippines-jokowi-duterte-authoritarianism/} accessed 29 June 2020.

84 Farnaz Fassihi, ‘Power struggle hampers Iran's coronavirus response’, *New York Times* (2020), available at: {https://www.nytimes.com/2020/03/17/world/middleeast/coronavirus-iran-rouhani.html} accessed 29 June 2020.

85 Riaz Hassan, ‘Pakistan's civil-military relations’, *Yale Global* (2020), available at: {https://yaleglobal.yale.edu/content/pakistans-civil-military-relations} accessed 29 June 2020; see also Ayesha Siddiqa, ‘Why is Pakistan spending so much money on defence amid COVID-19?’, *Aljazeera* (2020), available at: {https://www.aljazeera.com/indepth/opinion/pakistan-spending-money-defence-covid-19-200625115702999.html} accessed 29 June 2020.

86 Thusiyan Nandakumar, ‘Sri Lanka's militarised coronavirus containment has grave consequences’, *Medact* (2020), available at: {https://www.medact.org/2020/blogs/sri-lanka-coronavirus/} accessed 30 October 2020.

87 Frances Z. Brown et al., ‘How Will the Coronavirus Reshape Democracy and Governance Globally?’, Carnegie Endowment (2020), available at: {https://carnegieendowment.org/2020/04/06/how-will-coronavirus-reshape-democracy-and-governance-globally-pub-81470} accessed 29 June 2020.

88 See, for instance, ‘Coronavirus: Why are international comparisons difficult?’, *BBC* (2020), available at: {https://www.bbc.co.uk/news/52311014} accessed 30 October 2020; see also Norman Fenton et al., ‘Coronavirus: country comparisons are pointless unless we account for these biases in testing’, *The Conversation*, available at: {https://theconversation.com/coronavirus-country-comparisons-are-pointless-unless-we-account-for-these-biases-in-testing-135464} accessed 30 October 2020.

89 Kate Maclean, ‘Women leaders and coronavirus: Look beyond stereotypes to find the secret to their success’, *The Conversation* (2020), available at: {https://theconversation.com/women-leaders-and-coronavirus-look-beyond-stereotypes-to-find-the-secret-to-their-success-141414 accessed 29 July 2020.

90 Laura Spinney, ‘The coronavirus slayer! How Kerala's rock star health minister helped save it from Covid-19’, *The Guardian* (2020), available at: {https://www.theguardian.com/world/2020/may/14/the-coronavirus-slayer-how-keralas-rock-star-health-minister-helped-save-it-from-covid-19} accessed 29 July 2020.

91 President Nkurunziza denied the existence of the virus and kicked WHO official out of the country but subsequently died of Covid-related stroke, while his wife tested positive for the virus. See Paul Nantulya, ‘Post-Nkurunziza Burundi: The rise of the generals’, *Africa Centre* (2020), available at: {https://africacenter.org/spotlight/post-nkurunziza-burundi-the-rise-of-the-generals/} accessed 29 June 2020;

92 President Bolsonaro tested positive for COVID-19 after long denying the existence of the virus. See Teresa Bo, ‘COVID-19: Brazil deploys army to help protect Indigenous people’, *Aljazeera* (2020), available at: {https://www.aljazeera.com/news/2020/07/covid-19-brazil-deploys-army-protect-indigenous-people-200702085131273.html} accessed 29 June 2020.

93 President Trump spent the first six months of the pandemic denying the virus's existence or magnitude. See Ed Pilkington, ‘Six months of Trump's Covid denials: “It'll go away … It's fading”’, *The Guardian* (2020), available at: {https://www.theguardian.com/world/2020/jul/29/trump-coronavirus-science-denial-timeline-what-has-he-said} accessed 5 November 2020.

94 Sorcha Macleod, ‘Private security, human rights and COVID-19’, *European Journal of International Law* blog (2020), available at: {https://www.ejiltalk.org/private-security-human-rights-and-covid-19/} accessed 30 October 2020.

95 Pre-COVID-19 documented instances involved the Tamil Tigers, the Taliban, the Hezbollah, or the Zapatista movement, see Louis Lillywhite, ‘Non-State Armed Groups, Health and Healthcare’, Centre on Global Health Security Roundtable Summary, Chatham House (2015).

96 Ashley Jackson and Florian Weigand, ‘New pandemic, same old problems: introducing the Centre for the Study of Armed Groups’, Overseas Development Institute (2020), available at: {https://www.odi.org/blogs/17045-new-pandemic-same-old-problems-introducing-centre-study-armed-groups}accessed 29 July 2020; see also Mark Wilson, ‘The bizarre role of gangs during the coronavirus outbreak’, *Oxford Political Review* (2020), available at: {http://oxfordpoliticalreview.com/2020/07/19/the-bizarre-role-of-gangs-during-the-coronavirus-outbreak/} accessed 30 October 2020.

97 Clare Wenham et al., ‘Women are most affected by pandemics-lessons from past outbreaks’, *Nature* (2020), available at: {https://www.nature.com/articles/d41586-020-02006-z} accessed 29 July 2020; see also the Sex, Gender and COVID-19 project (ICRW), available at: {https://globalhealth5050.org/the-sex-gender-and-covid-19-project/} accessed 30 October 2020; and the United Nations Security Council Res 2439 (2018), available at: {http://unscr.com/en/resolutions/2439} accessed 30 October 2020.

98 A limited body of work acknowledges the potential gender impact of military health engagements in civilian populations; on the potential impact of military health delivery on maternal health outcomes, see Baringer and Heitkamp, ‘Securitizing global health’; on the silencing of gender inequalities and the ‘biomilitarism’ of the Zika virus response in Brazil, see Barbara Ribeiro et al., ‘Media coverage of the Zika crisis in Brazil: The construction of a “war” frame that masked social and gender inequalities’, *Social Science and Medicine*, 200 (2017), pp. 137–44; on gendered vulnerability and military-led securitisation in a postconflict setting, see Seckinelgin et al., ‘Securitization of HIV/AIDS in context’; for gender dynamics within the armed forces and military personnel, see Claire Duncanson and Rachel Woodward, ‘Regendering the military: Theorizing women's military participation’, *Security Dialogue*, 47:1 (2016), pp. 3–21.

99 See, for instance, Tony Kirby, ‘Evidence mounts on the disproportionate effect of COVID-19 on ethnic minorities’, *Lancet Respiratory Medicine*, 8:6 (2020), pp. 547–8.

100 Ifat Gazia, ‘In Kashmir, military lockdown and pandemic combined are one giant deadly threat’, *The Conversation* (2020), available at: {https://theconversation.com/in-kashmir-military-lockdown-and-pandemic-combined-are-one-giant-deadly-threat-142252} accessed 20 July 2020.

101 ‘UN: COVID-19 efforts hampered amid Israeli-Palestinian breakdown’, *Al-Jazeera* (2020), available at: {https://www.aljazeera.com/news/2020/07/covid-19-efforts-hampered-israeli-palestinian-breakdown-200722111602590.html} accessed 29 June 2020.

102 Interview with Francesco Checchi, ‘Reducing the toll of COVID-19 in Africa: What can be done?’, *LSHTM Viral* (2020), available at: {https://anchor.fm/lshtm/episodes/S1E21-Reducing-the-toll-of-COVID-19-in-Africa-What-can-be-done-ed2sp2} accessed 1 May 2020.

103 Ibid.

104 Maysoon Dahab et al., ‘COVID-19 control in low-income settings and displaced populations: What can realistically be done?’, *LSHTM News* (2020), available at: {https://www.lshtm.ac.uk/newsevents/news/2020/covid-19-control-low-income-settings-and-displaced-populations-what-can} accessed 29 June 2020.

105 Ibid.

106 Rene Loewenson, Kristen Accoe, Nitin Bajpai et al., ‘Reclaiming comprehensive public health’, *BMJ Global Health* (2020), pp. 1–6.

107 Rose Bernard and Gemma Bowsher et al., ‘Disinformation and epidemics: Anticipating the next phase of biowarfare’, *Health Security* (2020), pp. 1–10; see also Rose Bernard, Gemma Bowsher, and Richard Sullivan, ‘Cyber security and the unexplored threat to global health: A call for global norms’, *Global Security: Health, Science and Policy*, 5:1 (2020), pp. 134–41.

108 Gunhild Hoogensen Gjørv, ‘Coronavirus, Invisible Threats and Preparing for Resilience’, NATO (2020), available at: {https://www.nato.int/docu/review/articles/2020/05/20/coronavirus-invisible-threats-and-preparing-for-resilience/index.html}accessed 29 July 2020.

109 Ibid.

110 For metaphor of assemblages linking public and private security actors in LMICs, see Rita Abrahamsen and M. C. Williams, Security Beyond the State: Private Security in International Politics (Cambridge: Cambridge University Press, 2011); Philippe M. Frowd and Adam J. Sandor, ‘Militarism and its limits: Sociological insights on security assemblages in the Sahel’, Security Dialogue, 49:1–2 (2018), pp. 70–82.

111 This process (by which militaries become a default answer to all security issues) has been described in the US by Rosa Brooks, How Everything Became War and the Military Became Everything: Tales from the Pentagon (New York: Simon & Schuster, 2016), pp. 1–448.

112 See, for instance, ‘Lessons From the Military for COVID-time Leadership’, Mckinsey & Company (2020), available at: {https://www.mckinsey.com/industries/public-sector/our-insights/lessons-from-the-military-for-covid-time-leadership#} accessed 29 July 2020

113 Alexandre Christoyannopoulos, ‘Stop calling coronavirus pandemic a “war”’, *The Conversation* (2020), available at: {https://theconversation.com/stop-calling-coronavirus-pandemic-a-war-135486} accessed 29 July 2020.

114 Christoyannopoulos, ‘Stop calling coronavirus pandemic a “war”’; see also Ileana I. Diaz and Alison Mountz, ‘Intensifying fissures: Geopolitics, nationalism, militarism, and the US response to the novel coronavirus’, Geopolitics, 25 (2020), pp. 1–9.

115 ‘COVID-19 and Conflict: Seven Trends to Watch’, International Crisis Group (2020), available at: {https://www.crisisgroup.org/global/sb4-covid-19-and-conflict-seven-trends-watch} accessed 29 July 2020.

116 Graham, ‘The Armed Forces and COVID-19’.

117 Nick Childs, ‘Great-Power Competition and COVID-19’, International Institute for Strategic Studies (2020), available at: {https://www.iiss.org/blogs/military-balance/2020/05/great-power-competition-covid-19} accessed 29 July 2020.

118 ‘Coronavirus: Russia brings army doctors home from Italy’, *BBC* (2020), available at: {https://www.bbc.com/news/world-europe-52557426} accessed 29 July 2020.

119 US Department of Defense, ‘Pakistan Delivers COVID-19 Response Supplies as Gesture of Partnership’ (2020), available at: {https://www.defense.gov/Explore/Features/Story/Article/2203676/pakistan-delivers-covid-19-response-supplies-as-gesture-of-partnership/} accessed 29 July 2020.

120 US Department of Defence, Operation Warp Speed (2020), available at: {https://www.defense.gov/Explore/Spotlight/Coronavirus/Operation-Warp-Speed/} accessed 4 November 2020.

121 Stockholm Peace and Research Institute predicts a drop in military expenditures after a significant increase in 2019; see ‘SIPRI Global Military Expenditure’ (2020), available at: {https://www.sipri.org/media/press-release/2020/global-military-expenditure-sees-largest-annual-increase-decade-says-sipri-reaching-1917-billion} accessed 29 July 2020.

122 On redefinitions of military doctrine for global health, see Falconer Hall, Horne, and Ross, ‘Comparison between defence healthcare engagement and humanitarian assistance’; on inadequacy of humanitarian guidelines for civil-military interactions, see Kamradt-Scott et al., ‘Saving Lives’; see also Balcius, ‘Maritime Military Humanitarian Civic Assistance Missions’.

123 For instance, in a Cambodian proposed emergency law, see Aun Chhengpor, ‘Skepticism rife following introduction of “State of Emergency” draft law’, *Voa Cambodia* (2020), available at: {https://www.voacambodia.com/a/skepticism-rife-following-introduction-of-state-of-emergency-draft-law/5355113.html} accessed 20 July 2020.

124 On practices of security and control, see Katharine Hall Kindervater, ‘The emergence of lethal surveillance: Watching and killing in the history of drone technology’, Security Dialogue, 47:3 (2016), pp. 223–38.

125 On big technology corporations and surveillance, see, for instance, Naomi Klein, ‘Screen New Deal’ (2020), available at: {https://theintercept.com/2020/05/08/andrew-cuomo-eric-schmidt-coronavirus-tech-shock-doctrine//} accessed 30 October 2020.

126 ‘Coronavirus: Security forces kill more Nigerians than Covid-19’, *BBC* (2020), available at: {https://www.bbc.com/news/world-africa-52317196} accessed 29 June 2020.

127 Njal Hostmaelingen and Heidi Beate Bentzen, ‘How to operationalise human rights for COVID-19 measures’, BMJ Global Health, 5:003048 (2020), pp. 1–410.1136/bmjgh-2020-003048PMC1057776132646855.

